# Context-Dependent Plastic Response during Egg-Laying in a Widespread Newt Species

**DOI:** 10.1371/journal.pone.0136044

**Published:** 2015-08-20

**Authors:** Zoltán Tóth

**Affiliations:** Lendület Evolutionary Ecology Research Group, Plant Protection Institute, Centre for Agricultural Research, Hungarian Academy of Sciences, Budapest, Hungary; Louisiana State University & LSU AgCenter, UNITED STATES

## Abstract

Previous research on predator-induced phenotypic plasticity mostly focused on responses in morphology, developmental time and/or behaviour during early life stages, but the potential significance of anticipatory parental responses has been investigated less often. In this study I examined behavioural and maternal responses of gravid female smooth newts, *Lissotriton vulgaris*, in the presence of chemical cues originating from invertebrate predators, *Acilius sulcatus* water beetles and *Aeshna cyanea* dragonfly larvae. More specifically, I tested the extent of oviposition preference, plasticity in egg-wrapping behaviour and plasticity in egg size when females had the possibility to lay eggs at oviposition sites with and without predator cues during overnight trials. I found that individuals did not avoid laying eggs in the environment with predator cues; however, individuals that deposited eggs into both environments adjusted the size of the laid eggs to the perceived environment. Females deposited larger eggs earlier in the season but egg size decreased with time in the absence of predator cues, whereas individuals laid eggs of average size throughout the investigated reproductive period when such cues were present. Also, egg size was found to be positively related to hatching success. Individuals did not adjust their wrapping behaviour to the presence of predator cues, but females differed in the extent of egg-wrapping between ponds. Females’ body mass and tail depth were also different between ponds, whereas their body size was positively associated with egg size. According to these results, female smooth newts have the potential to exhibit activational plasticity and invest differently into eggs depending on temporal and environmental factors. Such an anticipatory response may contribute to the success of this caudate species under a wide range of predator regimes at its natural breeding habitats.

## Introduction

Phenotypic plasticity, the ability of a genotype to produce alternative phenotypes in different environments, is an important concept in modern evolutionary thinking [1], [2], and there is an unceasing scientific interest to evaluate its potential role in generating and maintaining adaptive phenotypic variation in natural populations [3–8]. In aquatic organisms (e.g. freshwater cladocerans, molluscs, fish and amphibians), predator-induced defences have been studied extensively in pursuits of testing various model predictions related to the evolution of plasticity [9–15]. The majority of these studies examined plasticity during embryonic or larval development, and showed that individuals in early life history stages are often able to change their morphology, physiology and/or behaviour in order to be better adapted against predators. A less frequently considered evolutionary outcome is that individuals, if having the necessary cognitive capacity and neuro-endocrine prerequisites, may adjust their behaviour to the current environment irrespective of or in interaction with their past experience, i.e. exhibit a certain level of activational plasticity [8]. Alternatively, individuals may display spatial niche choice to reduce realized variation in their environment [7]. Such adaptive behavioural responses are especially relevant in species where individuals are likely to encounter different environments during their lifetime, i.e. the prevalent environmental variation is ‘fine-grained’ [16]. Any of these adaptations can contribute to the maximization of fitness by increasing individuals’ survival, and in case of reproducing adults, also that of their offspring. It is still poorly understood how parents exhibiting such responses may mitigate or counteract negative environmental conditions and, in fact, can be a potential reason for why transgenerational parental effects were found to be generally weak by a recent meta-analysis [17]. Cues by which individuals can reliably predict the future post-natal environment are of uttermost importance in any forms of anticipatory responses [18], [19]; however, this requirement of predictability may not be fulfilled in many systems under natural circumstances [17], [20].

In theory, egg-laying caudates (Amphibia: Salamandridae) can enhance their offspring’s survival by exhibiting anticipatory responses when non-lethal chemical cues from predators of their offspring are present. First, females may show site preference during oviposition and deposit their eggs into microhabitats where predation risk is perceived to be relatively low. So far, there is limited support for such spatial niche choice in newts. For instance, Orizaola & Braña [21] studied oviposition preference in four newt species (formerly all belonging to the *Triturus* genus) and found that only female marbled newts, *Triturus marmoratus*, differentiated between oviposition sites in response to chemical cues of a fish predator. In alpine newts, *Ichthyosaura alpestris*, Kurdíková et al. [22] found that females preferred to deposit their eggs on a particular temperature along a thermal gradient and irrespective of the vicinity of a predatory beetle, thus individuals maximized their own reproductive performance rather than offspring fitness. Second, females may exhibit plasticity in parental care and invest more into related behaviours in hazardous habitats. In many newt species, females wrap submerged plant materials around their eggs using their hind limbs [23]. Egg-wrapping has previously been associated with various environmental factors and found to provide protection against invertebrate predators and adult newts [24–25], mechanical damage [26], toxic pollution [27] and harmful UV radiation [28]. Although both between- and within-species variation in egg-wrapping is known to exist in caudates [25], [29–31], no study has experimentally tested what environmental cues may moderate the extent of egg-wrapping. Third, females may adjust their offspring’s phenotype according to their own environment or phenotype. These so called maternal effects, various forms of transgenerational plastic responses [32–34], include (at least in oviparous species) changing the composition or increasing the size of the deposited eggs when predation risk of the offspring is high [35–37]. Larger eggs are known to provide greater fitness to the offspring [38] (but see [39]), and are hypothesized to be effective against predators if it results in the enhanced growth, reduced foraging activity and/or increased reaction distance to predators of the hatched progeny [40]. So far, a handful of studies found evidence for this form of maternal effect in fish and amphibians [18], [35], [36], [41], but we still lack information about the generality of its occurrence in these vertebrate taxa.

As previous research on phenotypic plasticity primarily focused on developmental plasticity (i.e. different developmental trajectories triggered by environmental cues, which result in adaptive phenotypic variation [7]) in most amphibian model systems [42–51], we have limited information about how anticipatory parental behaviours are expressed and jointly contribute to individuals’ reproductive success (but see [52], [53]). It is fair to assume that within- and transgenerational plasticity may act and shape phenotypic evolution together, so studying these effects simultaneously should be of great interest in evolutionary biology [54], [55]. In this study I examined behavioural and maternal responses of gravid female smooth newts, *Lissotriton vulgaris*, in the presence of chemical cues originating from invertebrate predators. Specifically, I aimed to investigate the extent of oviposition preference, plasticity in egg-wrapping behaviour and plasticity in egg size when females had the possibility to lay eggs at oviposition sites with and without predator cues. Moreover, I also tested if any of these responses had an effect on hatching success of the deposited eggs, thus evaluate their fitness consequences during embryonic development. Olfactory cues are known to be important in predation risk assessment in aquatic environments [56], [57], and most likely serve as the basis for predator-induced behavioural responses in larvae [47] and kin recognition among conspecifics [58], [59] in this species. I hypothesized that females may use such cues either to avoid hazardous environments during egg-laying or to exhibit activational plastic responses in order to increase their offspring’s survival. Plasticity is expected to be facilitated by natural selection if populations inhabit a (spatially or temporally) heterogeneous landscape and gene flow among these populations hinders the evolution of local genetic adaptation [60], [61]. As all females originated from natural breeding sites where environmental conditions were expected to favour the evolution of plasticity according to *a priori* information, I predicted that females were likely to show some forms of activational plastic responses in the presence of predator cues during reproduction.

## Materials and Methods

### Study species and area

The smooth newt (*Lissotriton vulgaris*) is the most widespread newt species in Europe [62], and also one of the most successful salamandrids: despite the current population decline of many amphibians, smooth newts are very common and maintain stable populations across much of their range [63]. Smooth newts breed in a wide variety of freshwater habitats including slowly moving shallow waters, permanent ponds and temporary pools [23]. Females are philopatric and display strong pond fidelity, usually returning to their natal pond to reproduce [64]. The breeding period starts in early spring and lasts several months, during which females continuously deposit their eggs one-by-one, wrapping leaves or other plant material around them, up to 2–300 in total within one reproductive season [23].

Smooth newts regularly breed in permanent and semi-permanent ponds located on an approx. 10 km^2^ area of deciduous forests and natural clearings in the north-eastern part of the Pilis Mountains, Hungary [31], [59]. Animals for the experiment were collected from 4 focal ponds representing 3 groups of ponds (average distance among groups [calculated from the distances between the closest two ponds in two different groups; mean ± SD]: 1644.4 m ± 544.0; within-group average distance among ponds: 307.3 m ± 237.9; number of ponds in the groups: 2–3). A pond survey conducted by Bókony et al. (unpublished data) in the study area in 2014 provided detailed information about the abundance of predatory invertebrates that can prey upon newt eggs and larvae in some of the ponds (S1 Table). Preliminary results showed that the weighted abundance of potential predators differed significantly among ponds, and even between pairs of ponds within the same group in one out of the three cases (S1 Table). This suggests that the criterion of environmental heterogeneity in terms of predation pressure is likely to be fulfilled in the study area. Besides, all focal ponds had at least one adjacent pond within 363 metres (226.2 ± 130.7). According to an estimation of isolation by distance between populations of smooth newts in another study [65], adult migration rate can be expected to be approx. 2% between the focal and adjacent ponds. Because of that, gene flow at least between these ponds may also reach a sufficient level to facilitate the evolution of plastic responses [60]. Number of collected individuals and coordinates of the focal ponds are shown in Table 1.

**Table 1:** Coordinates of different ponds and the total number of animals collected for the experiment. Focal ponds are shown in bold.

Ponds	Coordinates	Collected animals	Number of collected individuals
**Alsóhosszúréti-tó (‘pond A’)**	**47°42’55” N, 19°01’23” E**	**Focal female smooth newts (*Lissotriton vulgaris*)**	**24**
**Felsőhosszúréti-tó (‘pond F’)**	**47°43’36” N, 19°00’58” E**	**Focal female smooth newts**	**13**
**Ilona-tó (‘pond I’)**	**47°42’48” N, 19°02’25” E**	**Focal female smooth newts**	**23**
**Katlan (‘pond K’)**	**47°42’42” N, 19°02’40” E**	**Focal female smooth newts**	**20**
Pond near Pilisjászfalu	47°38’41” N, 18°46’31” E	Additional smooth newts	6 (3 pairs)
		Adult lesser diving beetles (*Acilius sulcatus*)	20
		Agile frog (*Rana dalmatina*) eggs	~300
small pond near Bajna	47°38’41” N, 18°36’41” E	Southern hawker (*Aeshna cyanea*) larvae	14
		Adult lesser diving beetles (*Acilius sulcatus*)	7

### Animal collection and housing

Gravid female smooth newts were captured by dip-net from the focal ponds from 17^th^ March to 14^th^ April, 2014. During the above period, 16 animals (0–8 from each pond per week) were caught and transported to the laboratory of the Experimental Station Júliannamajor of the Plant Protection Institute, Centre for Agricultural Research, Hungarian Academy of Sciences, on the first day of each week. Females were housed separately in transparent plastic containers (30 (L) × 20 (W) × 12 (H) cm), filled with approx. 3 litres of aerated reconstituted soft water (RSW [66]). Dry beech leaves were also provided for shelter and climbing surface. Animals were kept under a 13(L): 11(D) photoperiod at 16.7 ± 0.8°C ambient temperature, and fed *ad libitum* with live *Tubifex* worms throughout their captivity (except for the experimental trials; see below). On the 2^nd^ day of the week, half of the females were randomly selected for the first experimental trial (i.e. on the 2^nd^ day of their captivity), which took place in separate testing containers and lasted 11 hours during the night; the other half of the females were tested two days later on the 4^th^ day of the week (i.e. on the 4^th^ day of their captivity; second experimental trial). After the two trials, individuals were placed back to their housing container. On the 5th day of the week, I anesthetized the animals by inserting them into a 0.2% solution of MS-222 (CAS: 886-86-2, Sigma-Aldrich Co., USA), and took photographs of the animals using a Canon Powershot SX50 HS digital camera (Canon Inc., Japan), measured their body mass (± 0.01 g) and injected visible elastomer tags (2–3 mm long purple stripes; Northwest Marine Technology Inc., USA) under their tail skin for individual recognition. This latter procedure helped to avoid capturing the same animal more than once at the ponds. Water in the housing containers was also changed on the 5^th^ day. Females were kept in the lab for an additional two days, and then released at the site of capture on the first day of the next week, when another 16 females were captured at the same four locations. Using this scenario, I collected 80 females in total in 5 consecutive weeks during the spring.

Invertebrate predators were collected by dip-net between 5^th^ and 13^th^ March, 2014 from three ponds near the study area in Hungary. I captured adult *Acilius sulcatus* (Coleoptera: Dytiscidae) water beetles (body mass [mean ± SD] measured at the end of the experiment: 323.6 ± 40.1 mg) and southern hawker, *Aeshna cyanea* (Odonata: Aeshnidae) larvae (instars F1-F3; body mass: 959.5 ± 93.7 mg) and used them for producing predator cues during the experimental trials. The number of collected individuals and coordinates of the ponds are shown in Table 1. Water beetles were kept in threes in plastic boxes filled with approx. 2 litres of RSW, whereas dragonfly larvae were housed individually in plastic cups filled with approx. 0.2 litres of RSW. The plastic boxes also contained beech leaves for shelter to the beetles, while wooden sticks were provided to the *Aeshna* larvae as perching sites. Individuals, outside the trials, were fed *ad libitum* with *Tubifex* worms, and their water was changed at least once a week.

I also captured 3–3 male and female smooth newts using dip-net on 5^th^ and 6^th^ March, 2014 in one of the ponds (Table 1). Animals were kept in pairs in plastic containers filled with approx. 6 litres of RSW and equipped with *Elodea* threads for egg laying and clay pots for hiding and climbing. From 8^th^ March, all three females started laying eggs on the plants, which eggs were later used for feeding the predatory beetles during the experimental trials. Eggs were collected every third day by cutting the *Elodea* leaves on which they were deposited and then carefully unwrapped them if necessary (note that *Acilius sulcatus* water beetles have previously been found to consume unwrapped eggs at a higher proportion [25]). Afterwards, eggs were kept in RSW (barely covering the eggs) at 10°C to slow down their development. Additionally, one clutch of approx. 300 agile frog, *Rana dalmatina*, eggs was also collected from the same pond. Hatched tadpoles were housed together in a plastic container filled with 20 L of RSW and fed *ad libitum* with boiled spinach. These tadpoles were used for feeding the dragonfly larvae during the experimental trials.

Eggs deposited by the focal females during the experimental trials were also collected together with the *Elodea* leaves. After unwrapping (if necessary), photographs were taken of the eggs deposited into each of the two tested environments (see below), and then kept at 17.2 ± 0.8°C ambient temperature in small plastic boxes filled with approx. 0.1 litre of RSW. During the whole procedure, eggs deposited into different environments were treated and kept separately for each female. Eggs were checked every day and non-developing or mouldy eggs were removed, until all larvae hatched. By 12^th^ May, all animals including predators, additional smooth newt pairs and hatched larvae of the tested females were released at the site of capture.

### Ethics statement

The study area belongs to the operational area of the Danube-Ipoly National park, Hungary. All animals were captured by dip-net at the site of collection (Table 1) and then brought to the laboratory in individual plastic boxes appropriate for transportation. The experiment reported in this paper complies with current laws on animal experimentation in Hungary and the European Union. This study was approved by the institutional ethics committee (Hungarian Academy of Sciences, Centre for Agricultural Research, Plant Protection Institute, Institutional Animal Care and Use Committee; MTA ATK NÖVI MÁB) in accordance with Good Scientific Practice guidelines and national legislation. All sampling procedures and experimental manipulations of this study were reviewed and specifically approved by the national authority of the Middle-Danube-Valley Inspectorate for Environmental Protection, Nature Conservation and Water Management, who issued the permission to capture (KTF: 603-3/2014) and conduct experiment on the animals (KTF: 603-4/2014).

### Experimental procedure

During the experimental trials, females were put individually into a testing container (60 (L) × 40 (W) × 17 (H) cm) filled with approx. 17 litres of RSW. Each of these containers had two compartments, which were created by gluing an 18.5 (L) × 15 (W) cm plastic plate along the long central axis of the container (S1 Fig). Into each of these two compartments (representing one fourth of the inner area of the container), a standard infusion tube could be inserted through the wall, one tube leading from a ‘no predator-cue’ container (i.e. containing only RSW) and another tube leading from a ‘predator-cue’ container (containing RSW in which invertebrate predators were kept; see below). Each testing container had its own ‘no predator-cue’ and ‘predator-cue’ container throughout the experiment (S2 Fig). Out-flow water left each testing container through a 10 mm diameter plastic tube, which was affixed into the middle of the wall at the opposite end of the container (~70 mm from the bottom). During the trials, water from the ‘no predator-cue’ and ‘predator-cue’ containers flowed to the testing container due to the force of gravitation arose from the difference in relative elevation between the testing container and the associated ‘no predator-cue’ and ‘predator-cue’ containers. Prior to the experiment, infusion tubes were standardized to have a 0.64 ± 0.09 ml/s flow rate, which enabled continuous in-flow for 11 hours. All infusion tubes were tested five times for consistent flow rate prior to the experiment (mean SD within tubes: 0.006 ± 0.003 ml/s).

At 16:00 one day before the trial, ‘predator-cue’ containers were filled with 30 litres of RSW, and one dragonfly larva and two water beetles (from the same housing box) were put into each of these containers in predator cages, each species separately. Predator cages were made of an opaque plastic tube with 11 cm diameter and covered by nets on both ends. On the day of the trial, ‘no predator-cue’ containers were also filled with 30 litre of RSW, while the predators were fed at 18:00 in the ‘predator-cue’ containers. Each dragonfly larva obtained 120–140 mg of agile frog, *Rana dalmatina*, tadpoles (2–4 tadpoles; the required number was adjusted each week), whereas the two water beetles in each predator cage were fed with 3 smooth newt eggs (98.8 ± 11.2 and 83.3 ± 30.0% of the offered prey was consumed by the predators, respectively, by the end of the trials). At 19:00, a 14 cm long *Elodea* thread was affixed to the container’s wall in each compartment, 3 cm far from the in-flow source. Then, the infusion tubes were inserted to their place in each compartment, and the water from the ‘no predator-cue’ and ‘predator-cue’ containers started to flow. At 20:05, eight randomly chosen females were put into their randomly selected testing containers (where the outflow tubes were inserted; S1 Fig). Then, each container was covered by transparent Plexiglass for the duration of the trial. Each trial lasted from 20:15 to 07:15 next day (i.e. 11 hours during the night). After collecting the eggs deposited onto the plants in each compartment, females were put back to their appropriate housing container. The testing containers were emptied, washed thoroughly and cleaned using 70% ethanol to remove all potential remnants of chemical cues. Predators were rotated between containers after the trials to minimize error arising from differences between individual predators.

### Statistical analysis

All analyses were performed in R 3.1.2 [67]. Only those females were analysed that deposited eggs at least in one of the two environments during the experimental trials (68 out of 80 animals). For these animals, I measured the snout-to-vent length (SVL, henceforward), tail length and tail depth from the digital images using ImageJ 1.48v (US National Institutes of Health, USA [68]). Similarly, length along the long axis of symmetry and the largest width were measured on all eggs, which were deposited by these females during the trials (note that the jelly coat around the eggs has an oval shape [23]). I performed principal component analyses (PCA) on each female’s average egg length and width, and considered the first principal component as a proxy for egg size in the subsequent analyses. The first component (egg size, henceforward) was positively correlated with both length measures, i.e. higher values indicated eggs with wider and longer jelly coat, and accounted for 88% of the total variance.

I tested for any effect of pond of origin on overall morphology using multivariate analysis of variance (MANOVA) with pond of origin, time of trial (expressed as the number of days passed from 1st of March to the day of the trial) and their interaction as potential predictors, and body mass, SVL, tail length, and tail depth as response variables. Then I evaluated the effect of pond on each morphological trait independently using linear models [69]; pond was included as a fixed factor into these models, whereas mass was a general body size covariate (except for the fitted model on mass itself) in order to be able to compare size-corrected morphology.

Oviposition preference was calculated for each individual as the number of eggs laid in the ‘no predator-cue’ environment divided by the total number of deposited eggs. I fitted generalized linear model (GLM) with quasi-binomial error distribution (to account for overdispersion), into which time of trial, SVL and pond of origin were included as potential predictors. Wrapping ratio was calculated as the number of wrapped eggs divided by the number of laid eggs in each environment (only for females which deposited eggs into both environments; *N* = 42). Here I fitted generalized linear mixed-effect models (GLMM) with binomial error distribution and Laplace approximation. SVL, time of trial, pond of origin and the interaction of the latter two with environment were added as fixed effects, whereas ‘Identity’ was included as a random factor into this model. Size of the eggs deposited into each environment was analysed for these female (*N* = 42) using linear mixed-effect model (LMM) with restricted maximum likelihood approximation. ‘Identity’ was included into this model as a random factor, and SVL, time of trial, pond of origin and the interaction of the latter two with environment as explanatory variables. Hatching success was computed for each individual (*N* = 42) as the number of hatched larvae divided by the number of deposited eggs in each environment. I investigated how oviposition preference, wrapping ratio and eggs size affected this dependent variable using a GLMM with binomial error distribution and Laplace approximation. ‘Identity’ was included as a random factor. In this model, oviposition preference was calculated as the proportion of eggs laid in each of the two environments. In all model fitting I used backward removal procedure, starting with the full models containing all variables, and then dropped the predictor with the highest *P*-value in each step until only *P*≤0.05 effects remained (if there were any) in the final models. To estimate the significance of the potential predictors, I used *F*-tests in models with Gaussian and Wald *F*-test in the model with quasibinomial error distribution [70]. In all GLMMs, I used Wald *χ* ^2^ tests during model selection (which method is fast, but tends to overstate the importance of some effects) and applied parametric bootstrap with 2000 iterations to obtain a more precise estimation for the significance of each explanatory variable in the final models [71]. Requirements of the fitted models were checked by plot diagnosis. All tests were two-tailed with alpha set to 0.05. Data used in the above analyses is fully available from the figshare database (http://dx.doi.org/10.6084/m9.figshare.1291124).

## Results

### Morphological characteristics of the females from different ponds

Individuals from different ponds significantly differed from each other in their morphological traits (MANOVA, Wilks’ λ = 0.58, approx. *F*_12,159.04_ = 3.0, *P* = 0.001), whereas time of trial had a marginally non-significant effect (Wilks’ λ = 0. 68, approx. *F*_4,59_ = 2.4, *P* = 0.057). The interaction between time and pond did not affect these studied characteristics (Wilks’ λ = 0. 80, approx. *F*_12,148.45_ = 1.1, *P* = 0.391). In particular, I found significant differences in body mass (*F*_3,64_ = 7.20, *P*<0.001) and tail depth (*F*_3,62_ = 4.20, *P* = 0.009) between females from different ponds, whereas pond had no effect on SVL (*F*_3,63_ = 2.23, *P* = 0.093) or tail length (*F*_3,62_ = 1.85, *P* = 0.148). Time of trial was negatively related to tail depth (*F*_1,62_ = 8.97, *P* = 0.004), but did not affect other morphological traits (body mass: *F*_1,63_ = 0.19, *P* = 0.668; SVL: *F*_1,65_ = 0.39, *P* = 0.536; tail length: *F*_1,64_ = 2.08, *P* = 0.154). Mass was significantly related to all three length measurements (SVL: *F*_1,66_ = 133.04, *P*<0.001; tail depth: *F*_1,62_ = 51.19, *P*<0.001; tail length: *F*_1,65_ = 96.35, *P*<0.001). Mean ± SD for each morphological characteristic in the four ponds is shown in Table 2.

**Table 2:** Morphological characteristics (mean ± SD) of focal females that laid eggs at least in one of the tested environments during the trials (*N* = 68). SVL is the abbreviation for the snout-to-vent length.

	Pond A (*N* = 21)	Pond F (*N* = 12)	Pond I (*N* = 19)	Pond K (*N* = 16)
Body mass (in g)	1.52 ± 0.23	1.47 ± 0.22	1.24 ± 0.15	1.33 ± 0.21
SVL (in mm)	39.8 ± 1.9	40.1 ± 2.2	37.8 ± 1.9	37.8 ± 2.1
Tail length (in mm)	34.0 ± 2.5	34.3 ± 3.0	31.3 ± 2.5	31.4 ± 2.1
Tail depth (in mm)	6.4 ± 0.8	5.7 ± 0.7	5.4 ± 0.6	5.9 ± 0.7

### Females’ responses to the presence of predator cues

**Fig 1:**
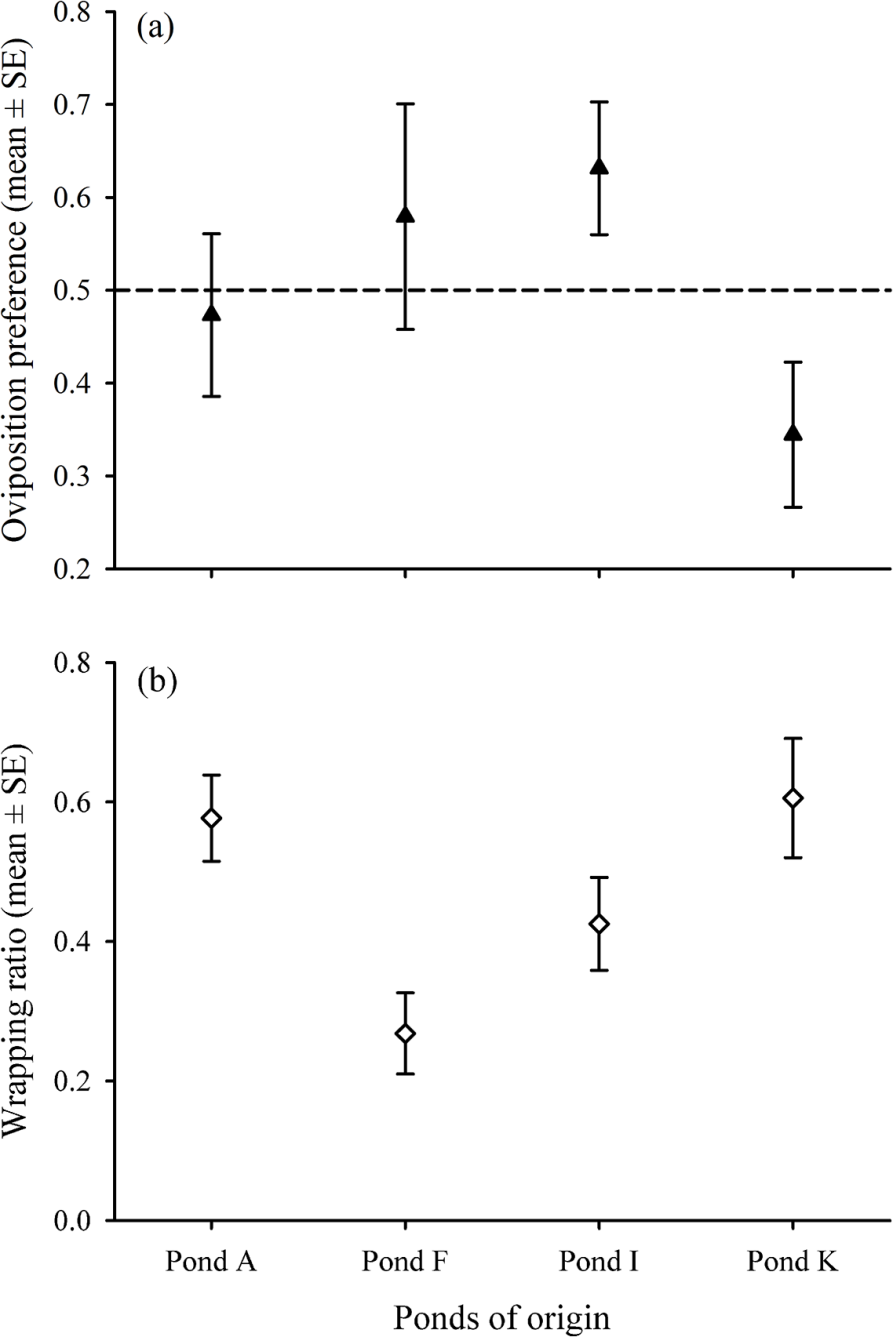
Oviposition preference (a) and wrapping ratio (b) in the focal ponds. Oviposition preference was calculated as the number of eggs deposited into the ‘no predator-cue’ environment divided by the total number of laid eggs, thus values greater than 0.5 represent a preference for the ‘no predator-cue’ environment (zero-preference is indicated by horizontal dashed line). Wrapping ratio was expressed as the proportion of wrapped eggs in each environment. In (a), difference among ponds is not significant despite the apparent deviation between ‘pond I’ and ‘pond K’; this difference diminished when only those females were included into the model which deposited eggs into both environments. In (b), the pooled data (i.e. for both environments) is shown.

**Fig 2:**
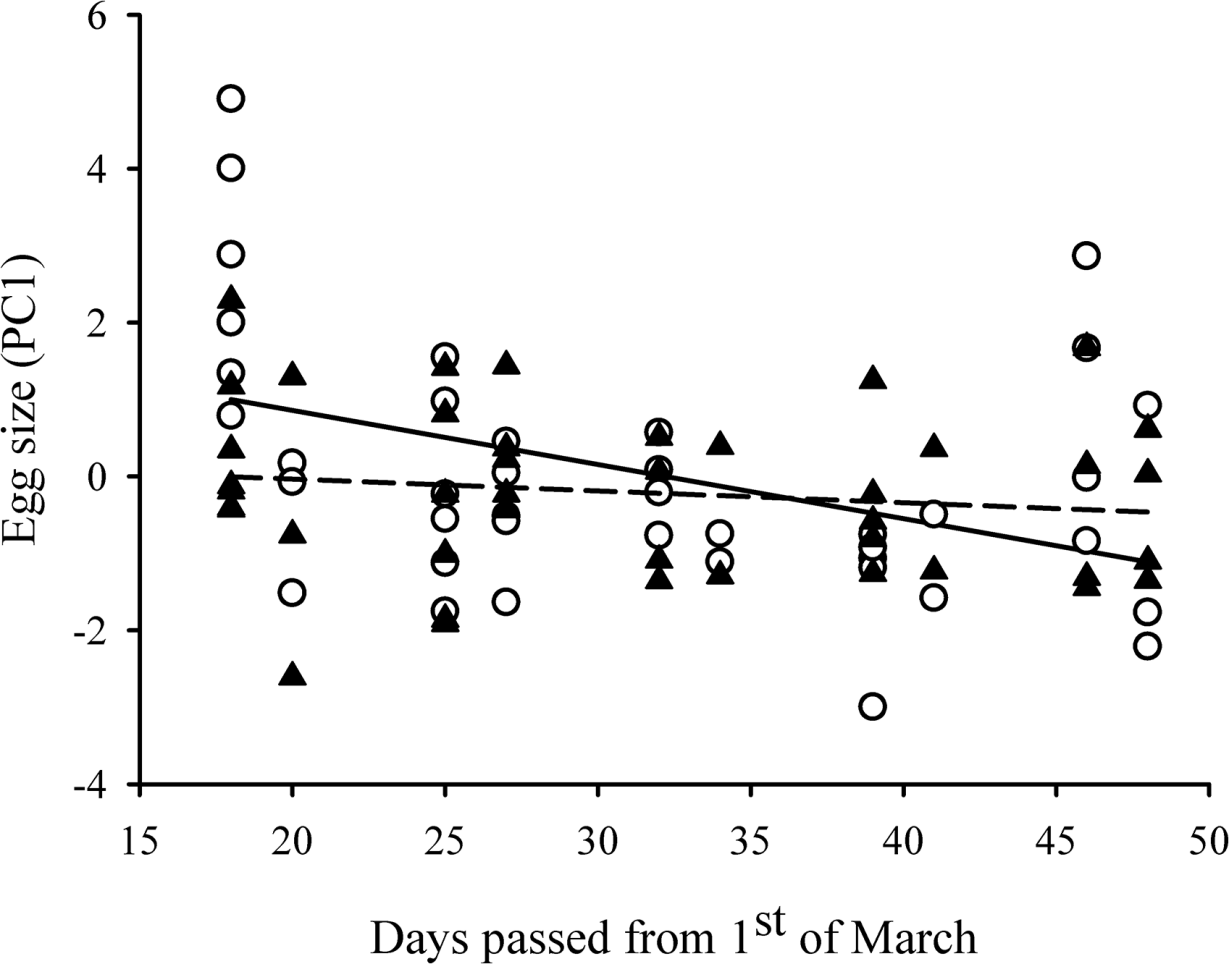
The effect of environment and time on egg size. Egg size is the first principal component derived from a PCA, which was performed on the average length and width of the jelly coat around the eggs deposited by each female in each environment. Open circles denote eggs laid into the ‘no predator-cue’ environment, whereas the black triangles represent eggs deposited into the ‘predator-cue’ environment. Regression lines indicate the changes in egg size with time in the ‘no-predator-cue’ (solid line) and the ‘predator-cue’ environment (dashed line), respectively.

Oviposition preference was not affected by any of the investigated predictors (all *P>*0.185; Table 3), although females originating from ‘pond I’ tended to avoid the ‘predator-cue’ environment during oviposition, whereas the opposite trend was found in females from ‘pond K’ (Fig 1A). Approximately one third of the animals laid eggs only into one environment (26 out of 68 females), but these females did not avoid the presence of predator cues during oviposition either (binomial test for the equality of probabilities: *N*_‘predator-cue’_ = 12, *N*_‘no predator-cue’_ = 14, *P* = 0.845). Wrapping ratio was found to be significantly different among ponds (*ΔD* = 12.08, *P* = 0.019; Table 3), suggesting that locally adaptive levels of wrapping ratio may exist in some of the ponds (Fig 1B). The odds of wrapping an egg in ‘pond A’ was 1.58 [95% CI: 0.87–2.85], i.e. there was 1.58 wrapped eggs for every unwrapped one in this pond. This value decreased by a factor of 0.19 [0.07–0.50] in ‘pond F’, 0.45 [0.19–1.05] in ‘pond I’, and 0.82 [0.34–2.08] in ‘pond K’, respectively. None of the other predictors had significant effect on wrapping ratio (all *P*>0.143; Table 3). Egg size was positively related to SVL (*F*_1,39_ = 6.32, *P* = 0.016), which implies that larger females laid larger eggs during the trials (parameter estimate ± 95% CI: 0.20 [0.05–0.34]). The interaction between time of trial and environment also had a significant effect on egg size (*F*_1,40_ = 7.33, *P* = 0.010): females produced larger eggs earlier in the season but egg size decreased with time in the ‘no predator-cue’ environment (by -0.06 [-0.10–-0.02] per time unit), while in the ‘predator-cue’ environment individuals laid eggs of intermediate size throughout the season (Fig 2). Pond of origin did not have any significant effect on egg size either in itself or interaction with environment (both *P*>0.201; Table 3).

**Table 3:** Test statistics and significance of the investigated predictors from the fitted models of females’ responses to the presence of predator cues. *F*-tests were used in models with Gaussian (LMM) and quasibinomial error distribution (GLM), whereas parametric bootstrap with 2000 iterations was applied in GLMMs. Final models are in bold; test statistics and *P*-values for the non-significant predictors were computed by including them one by one into the final models. Random effect is given in SD ± 95% confidence interval. *ΔD* is the likelihood ratio statistic and denotes the difference in deviances of the models fitted with and without the given predictor.

Model	Response variable	Random effect (‘Identity’)	Predictors	df	F	*ΔD*	P
**GLM**	**Oviposition preference**	**-**	**- (null model)**				
			Time	1,66	0.87	-	0.355
			SVL	1,66	0.68	-	0.411
			Pond	3,64	1.66	-	0.185
**GLMM**	**Wrapping ratio**	**0.81 [0.55–1.17]**	**Pond**	**-**	**-**	**12.08**	**0.019**
			SVL	-	-	0.89	0.387
			Time	-	-	0.14	0.732
			Environment	-	-	0.13	0.721
			Pond × Environment	-	-	0.78	0.826
			Time × Environment	-	-	2.27	0.143
**LMM**	**Egg size**	**0.73 [0.44–1.19]**	**SVL**	**1,39**	**6.32**	**-**	**0.016**
			**Time**	**1,39**	**9.19**	**-**	**0.004**
			**Environment**	**1,40**	**1.03**	**-**	**0.316**
			**Time × Environment**	**1,40**	**7.33**	**-**	**0.010**
			Pond	3,36	1.09	-	0.370
			Pond × Environment	3,37	1.62	-	0.201
**GLMM**	**Hatching success**	**2.09 [1.46–3.10]**	**Egg size**	**-**	**-**	**7.99**	**0.007**
			Oviposition preference*			1.18	0.296
			Wrapping ratio			0.08	0.790

*expressed here as the proportion of eggs laid in each of the two environments.

### Relationship between hatching success and the extent of plasticity in different traits

**Fig 3:**
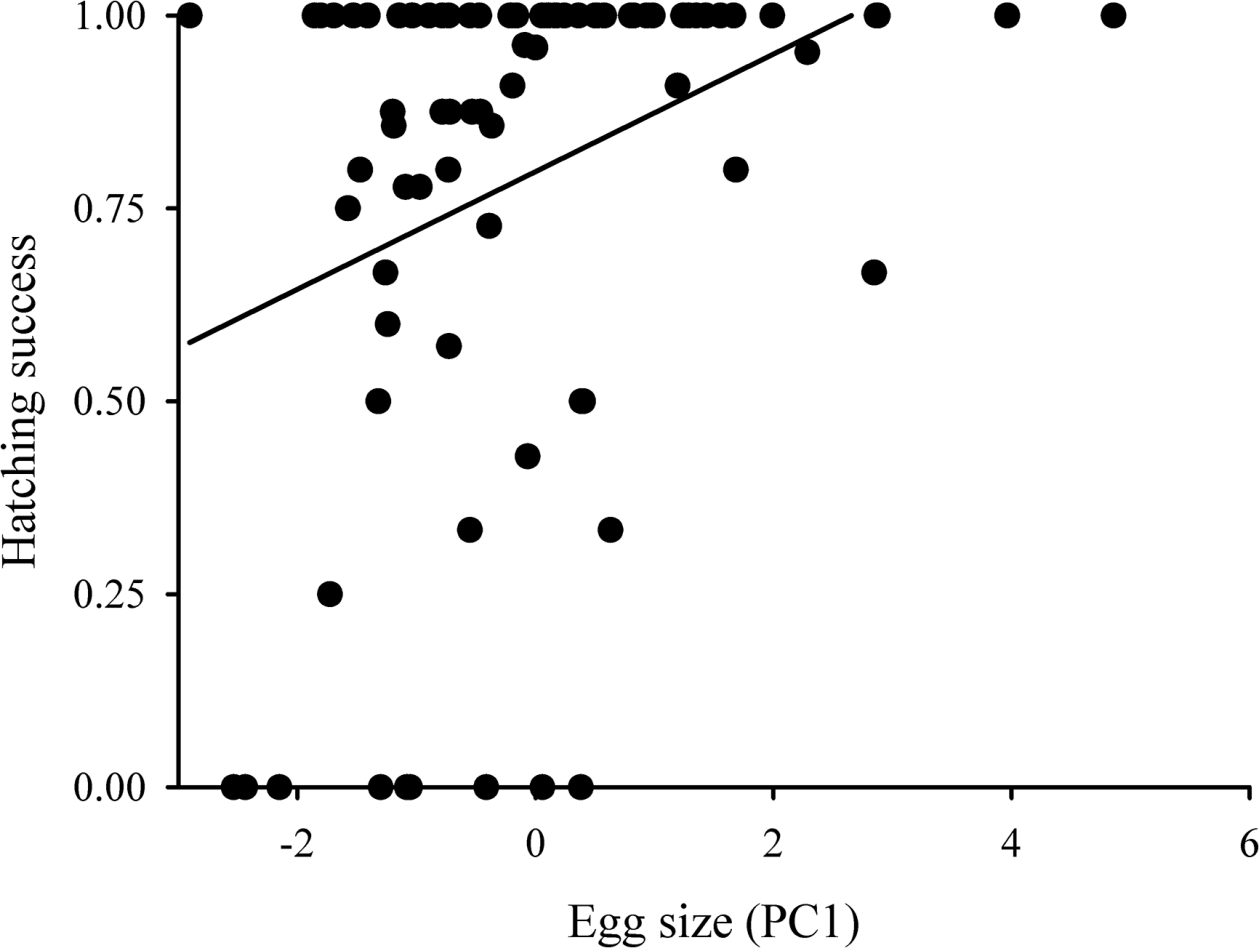
Relationship between hatching success and egg size. Hatching success was calculated as the proportion of hatched eggs in each environment. As environment had no effect on hatching success, the regression line is fitted to the pooled data.

Hatching success was positively related to egg size (*ΔD* = 7.99, *P* = 0.007; Fig 3), implying that larvae had higher chances of successful hatching from larger eggs than from smaller eggs. The odds of hatching was 13.76 [6.36–37.05], which increased by the factor of 1.92 [1.21–3.24] with each egg size unit. Other predictors had no effect on hatching success (all *P*>0.296; Table 3).

## Discussion

In this study, I investigated how gravid females of smooth newts adjusted their reproductive behaviour to the presence of predator cues, and thus exhibited anticipatory responses. I found no indication for substantial oviposition preference among individuals, but those animals that deposited eggs into both the ‘no predator-cue’ and the ‘predator-cue’ environments were found to adjust the size of their laid eggs to the perceived environment. Females laid larger eggs at the beginning of the season but egg size decreased with time during the reproductive season in the absence of predator cues, while individuals deposited eggs of intermediate size throughout the season when such cues were present. Besides, individuals did not adjust their wrapping behaviour to the presence of predator cues, but females differed in the extent of egg-wrapping between ponds of origin. Females’ body mass and tail depth were also different between ponds, whereas their body size (SVL) was positively associated with the size of the laid eggs. According to these results, female smooth newts have the potential to exhibit activational plasticity in the form of a maternal effect when chemical cues from predators are present, by which individuals may allocate their reproductive effort differently in less and more risky natal environments throughout the season.

Individuals did not display unequivocal oviposition preference during the tests, most probably due to the high variation between individuals in the proportion of eggs laid in the ‘no predator-cue’ environment, especially at two breeding sites. This finding is not surprising as such niche choice is less likely to be advantageous in small ponds where invertebrate predators are abundant (such as smooth newts’ typical breeding habitats in the study area), contrary to spatially more structured and/or large ponds, where predator-free microhabitats for egg-laying can potentially exist. Also, this result is in accordance with the majority of previous findings on other caudate species [21], [22]. Those individuals that laid eggs in both environments did not wrap more eggs during deposition in the ‘predator-cue’ environment than in the ‘no predator-cue’ environment, but the extent of individuals’ egg-wrapping differed between ponds. These results indicate that egg-wrapping is a behavioural trait that individuals do not adjust to the current environmental conditions, irrespective of the presence of predator cues in their environment. Although local adaptation in behaviour, including anti-predatory responses, has been found in a few freshwater species [72–74], it is expected to be rarely evolved due to the high flexibility of behavioural traits in general [75]. More likely, the duration of the trials (i.e. overnight) was too short to elicit substantial changes in the extent of egg-wrapping, so the measured values may actually represent what is adaptive in females’ ponds of origin. Alternatively, sensibility to predator cues may be limited to a given period in an early life stage, after which it becomes fixed even if the concentration of predator cues changes over time. Previous studies showed that larvae of many caudates (including this species) decreased their activity and/or showed morphological adaptations when raised in the presence of predator cues [46–48], but we have limited knowledge about how juveniles’ early experiences determine adult behaviour in most species. Finally, it may also be possible that the primary role of egg-wrapping is not providing protection against invertebrate predators, despite the indirect evidence presented by some of the earlier studies [24], [25].

I found that egg size decreased as the reproductive season progressed in the absence of predator cues, but remained at an intermediate level when predator cues were present. I propose that this is an activational plastic response, and may be related to an expected positive effect of egg size on the progeny’s size and performance [38], [76–78]. Context-dependent maternal effect has been found by previous studies [32], [79], also in response to the presence of predator cues [18], [35], [36], and expected to be favoured by natural selection if increased egg size has higher fitness benefits in hazardous environments than under optimal conditions [40], [80], [81]. Contrary to this prediction, the observed finding rather indicates that increased egg size in smooth newts provides provisionally higher benefit at the beginning of the reproductive season in less risky environments, which benefit diminishes as the season progresses. An intriguing explanation for this pattern is that ponds with no or low amount of predator cues may be more ephemeral. As the reproductive period advances there is a higher risk of desiccation at such temporal sites, leaving no time for the larvae to reach metamorphosis, thus females may invest less and less into the deposited eggs in such habitats. If so, the observed pattern can be adaptive and context-dependent resource allocation may allow females to maximize their offspring’s survival probabilities in different breeding environments. Alternatively, females that lay bigger eggs in the absence of predators were more likely to be captured at the beginning of the experiment, whereas females that lay typically smaller eggs in such environments were caught and tested later in the season, leading to the observed pattern in egg size. With the applied experimental design, it is not possible to discard either of these two potential explanations.

Female smooth newts are known to be strongly philopatric [64]; nevertheless, migration between ponds has been observed between reproductive seasons in the species [65]. Moreover, Weddeling et al. [82] also reported that adults can leave their current breeding site under suitable weather conditions (e.g. on rainy days), although such terrestrial activity and its consequences on reproductive investment was not examined in their study. Nevertheless, this observation implies that females may visit several ponds for egg-laying within a single breeding season, and thus encounter various aquatic environments. In that case, being able to adjust their reproductive effort to local conditions may be highly advantageous. Alternatively, benefits may arise if there is a temporal change in predator abundance in females’ natal pond during the season; eggs that are laid at the beginning of the season may face low predation risk and have–at the least–higher hatching success due to their larger size. It is not known whether or not a trade-off between egg size and egg number exists in smooth newts, but body size has been found to be a good predictor of both the size (this study) and number of eggs [83] females can deposit during reproduction (i.e. larger females can lay more and larger eggs).

Environmental predictability is a major, but often neglected, prerequisite in studies of anticipatory parental effects; such responses can only be favoured by natural selection if the parental environment sufficiently predicts the offspring environment [20]. In this study, I only assumed that this condition is met as predictability can generally be expected in environments that exhibit spatial variation, such as local differences in predation [17], [84]. Also, adaptivity of the observed maternal response has not been investigated explicitly, thus there is no information about how much this change in egg size can affect, for instance, hatching time, time to metamorphosis or larval survival. Nevertheless, I demonstrated that reproducing females are able to respond to the presence of predator cues. Although the influential effect of fine-grained environmental variation on phenotypic traits is predicted by theory, it has been rarely supported by experimental studies in amphibians. Future work should incorporate the experimental testing of the adaptive value of both maternal anticipatory and larval plastic responses in the presence of predator cues, and thus evaluate the long-term consequences of this maternal effect on phenotypic variance in smooth newts (for a similar approach see [85]).

In conclusion, results suggest that female smooth newts originating from the studied ponds have the ability of expressing a predator-induced, immediate plastic response in the form of a context-dependent maternal effect during reproduction. Females are likely to invest differently into egg production depending on temporal and environmental factors, which may contribute to the success of this caudate species under a wide range of predator regimes at its natural breeding habitats. Also, this study emphasizes the importance of investigating multiple phenotypic responses to explore the full repertoire of phenotypic adaptations by which individuals can cope with stress and variation in their environment.

## Supporting Information

S1 Fig Testing container with the two compartments (a,b), the adjusted infusion tubes (c,d), the small plastic tubes for anchoring the *Elodea* threads (e,f), and the outflow tube (g) with its opening (h) covered by a piece of mosquito net. (DOCX)

S2 Fig An example of the apparatuses, which were used in the overnight trials during the experiment. It consists of a testing container (A) and its adjacent ‘predator-cue’ (B) and ‘no predator-cue’ (C) containers with the infusion tubes. Into the ‘predator-cue’ container, two predator cages (D, E) were also inserted for separating the two water beetles and the dragonfly larva.
(DOCX)

S1 Table Weighted abundance of invertebrate predators in different ponds of the study area. Samples were collected using hollow pipes as quadrates and dip-nets. The number of individuals in each predator class was weighted according to the dangerousness of the given predator class to amphibian larvae (following Van Buskirk & Arioli 2005), then summed for each sample. Predators included Aeshnidae dragonfly larvae (weighting score 3), *Dytiscus marginalis* adults and larvae (weighting score 3), *Notonecta glauca* adults (weighting score 2), *Acilius sulcatus* adults and larvae (weighting score 1), *Libellula*-type dragonfly larvae (weighting score 1), *Dolomedes fimbriatus* juveniles (weighting score 1), Corixidae adults (weighting score 1) and Hirudinae adults (weighting score 1). The six ponds significantly differed from each other in the weighted abundance of invertebrate predators (Kruskal-Wallis test, *χ* ^2^_5_ = 24.06, *P*<0.001), even after the removal of pond A with its extremely high value (*χ* ^2^_4_ = 11.61, *P* = 0.020). Within the same group, there was also a significant difference between the two ponds in Group 1 (Wilcoxon rank sum test, *Z* = 3.48, *P*<0.001), but not in the other groups (both *P*>0.135). Unpublished data for the calculation of weighted abundances was kindly provided by V. Bókony, who conducted the pond survey in the study area in 2014.
(DOCX)

S2 Table Test statistics and significance of the investigated predictors from the same models fitted to the original measures of the eggs (length and width) as to the first component of PCA on egg size. Final models are in bold; test statistics and *P*-values for the non-significant predictors were computed by including them one by one into the final models. Random effect is given in SD ± 95% confidence interval. The estimated length of the eggs in the ‘predator-cue’ environment was 3.17 mm [3.10–3.25] and their estimated width was 2.21 mm [2.14–2.27] (with SVL and time of trial centered on their mean). Both measures increased with SVL (length: by 0.03 mm[0.01–0.06] per SVL unit, width: by 0.03 mm [0.004–0.05] per SVL unit), and both length and width of the eggs in the ‘no predator-cue’ environment also decreased with time compared to eggs in the ‘predator-cue’ environment (length: by -0.01 mm [-0.02–-0.003] per time unit, width: by -0.01 mm [-0.01–-0.001] per time unit).
(DOCX)
