# Correction: Context-Dependent Plastic Response during Egg-Laying in a Widespread Newt Species

**DOI:** 10.1371/journal.pone.0138163

**Published:** 2015-09-09

**Authors:** Zoltán Tóth

There is an error in Fig 3: The regression line in the published Fig 3 indicates a linear relationship between egg size and hatching success; however, a generalized linear mixed-effect model was used to analyze the relationship of these variables, which is not a linear one. Please see the correct Fig 3 here.

**Fig 3:**
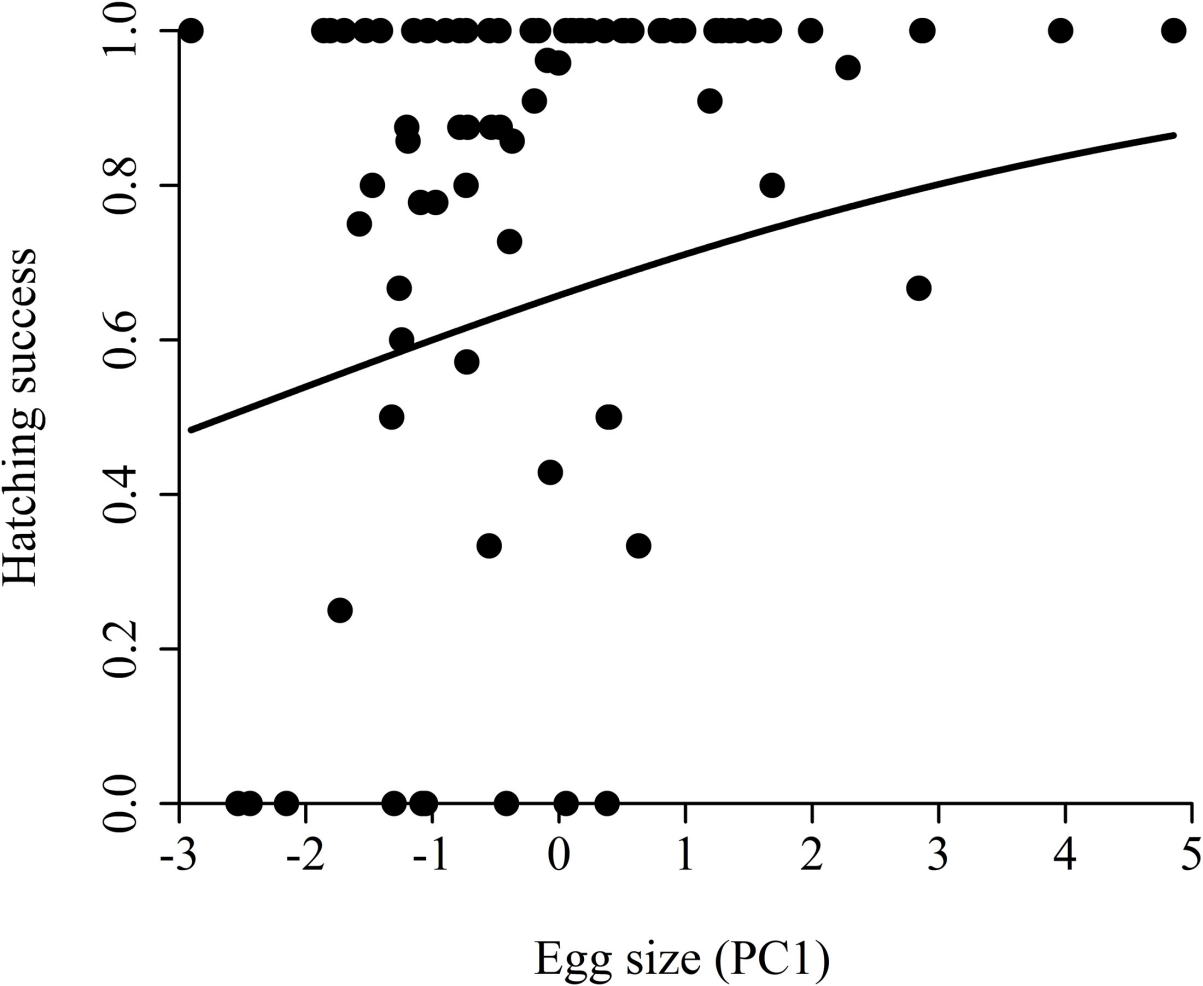
Relationship between hatching success and egg size. Hatching success was calculated as the proportion of hatched eggs in each environment. As environment had no effect on hatching success, the regression line is fitted to the pooled data.
