# Supplementary material for: Context-Dependent Plastic Response during Egg-Laying in a Widespread Newt Species
Source: PLoS One. 2015 Aug 20;10(8):e0136044. doi: 10.1371/journal.pone.0136044 (PMC4546198; doi:10.1371/journal.pone.0136044)

**S2 Figure.** Testing container with the two compartments (a,b), the adjusted infusion tubes (c,d), the small plastic tubes for anchoring the *Elodea* threads (e,f), and the outflow tube (g) with its opening (h) covered by a piece of mosquito net.


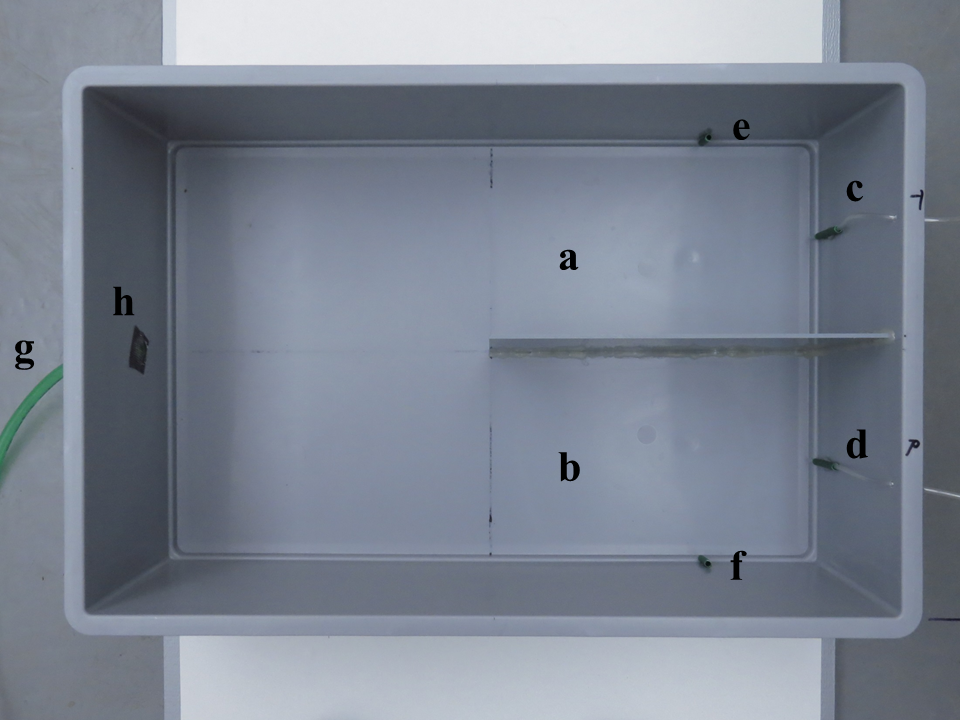

Supplement: S1 Fig — (DOCX) [file pone.0136044.s001.docx]
