# Supplementary material for: Context-Dependent Plastic Response during Egg-Laying in a Widespread Newt Species
Source: PLoS One. 2015 Aug 20;10(8):e0136044. doi: 10.1371/journal.pone.0136044 (PMC4546198; doi:10.1371/journal.pone.0136044)

**S3 Figure.** An example of the apparatuses, which were used in the overnight trials during the experiment. It consists of a testing container (A) and its adjacent ‘predator-cue’ (B) and ‘no predator-cue’ (C) containers with the infusion tubes. Into the ‘predator-cue’ container, two predator cages (D, E) were also inserted for separating the two water beetles and the dragonfly larva.


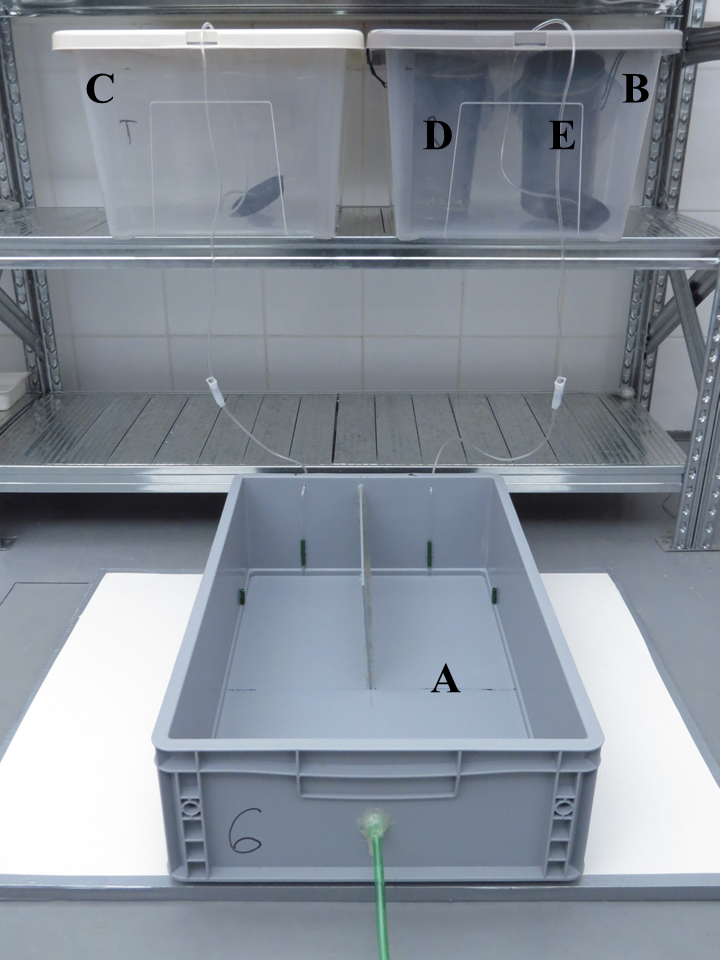

Supplement: S2 Fig — It consists of a testing container (A) and its adjacent ‘predator-cue’ (B) and ‘no predator-cue’ (C) containers with the infusion tubes. Into the ‘predator-cue’ container, two predator cages (D, E) were also inserted for separating the two water beetles and the dragonfly larva. (DOCX) [file pone.0136044.s002.docx]
