# Supplementary material for: Context-Dependent Plastic Response during Egg-Laying in a Widespread Newt Species
Source: PLoS One. 2015 Aug 20;10(8):e0136044. doi: 10.1371/journal.pone.0136044 (PMC4546198; doi:10.1371/journal.pone.0136044)
Supplement: S1 Table — Samples were collected using hollow pipes as quadrates and dip-nets. The number of individuals in each predator class was weighted according to the dangerousness of the given predator class to amphibian larvae (following Van Buskirk & Arioli 2005), then summed for each sample. Predators included Aeshnidae dragonfly larvae (weighting score 3), Dytiscus marginalis adults and larvae (weighting score 3), Notonecta glauca adults (weighting score 2), Acilius sulcatus adults and larvae (weighting score 1), Libellula-type dragonfly larvae (weighting score 1), Dolomedes fimbriatus juveniles (weighting score 1), Corixidae adults (weighting score 1) and Hirudinae adults (weighting score 1). The six ponds significantly differed from each other in the weighted abundance of invertebrate predators (Kruskal-Wallis test, χ 2 5 = 24.06, P<0.001), even after the removal of pond A with its extremely high value (χ 2 4 = 11.61, P = 0.020). Within the same group, there was also a significant difference between the two ponds in Group 1 (Wilcoxon rank sum test, Z = 3.48, P<0.001), but not in the other groups (both P>0.135). Unpublished data for the calculation of weighted abundances was kindly provided by V. Bókony, who conducted the pond survey in the study area in 2014. (DOCX) [file pone.0136044.s003.docx]

**S1 Table.** Weighted abundance of invertebrate predators in different ponds of the study area. Samples were collected using hollow pipes as quadrates and dip-nets. The number of individuals in each predator class was weighted according to the dangerousness of the given predator class to amphibian larvae (following Van Buskirk & Arioli 2005), then summed for each sample. Predators included Aeshnidae dragonfly larvae (weighting score 3), *Dytiscus marginalis* adults and larvae (weighting score 3), *Notonecta glauca* adults (weighting score 2), *Acilius sulcatus* adults and larvae (weighting score 1), *Libellula*-type dragonfly larvae (weighting score 1), *Dolomedes fimbriatus* juveniles (weighting score 1), Corixidae adults (weighting score 1) and Hirudinae adults (weighting score 1). The six ponds significantly differed from each other in the weighted abundance of invertebrate predators (Kruskal-Wallis test, *χ*^2^_5_=24.06, *P*<0.001), even after the removal of pond A with its extremely high value (*χ*^2^_4_=11.61, *P*=0.020). Within the same group, there was also a significant difference between the two ponds in Group 1 (Wilcoxon rank sum test, *Z*=3.48, *P*<0.001), but not in the other groups (both *P*>0.135). Unpublished data for the calculation of weighted abundances was kindly provided by V. Bókony, who conducted the pond survey in the study area in 2014.

| **Group of ponds** | **Ponds** | **Number of samples** | **Weighted abundance per sample (mean ± SD)** |
| --- | --- | --- | --- |
| Group 1 | Pond A | 11 | 25.73 ± 15.86 |
|  | János-tó | 14 | 1.64 ± 1.74 |
| Group 2 | Pond F | 10 | 5.3 ± 5.62 |
|  | Felsőhosszúrét-középső | 10 | 2.9 ± 2.77 |
| Group 3 | Pond I | 10 | 7.1 ± 5.7 |
|  | Mélymocsár | 12 | 4.17 ± 3.79 |
