# Supplementary material for: Context-Dependent Plastic Response during Egg-Laying in a Widespread Newt Species
Source: PLoS One. 2015 Aug 20;10(8):e0136044. doi: 10.1371/journal.pone.0136044 (PMC4546198; doi:10.1371/journal.pone.0136044)
Supplement: S2 Table — Final models are in bold; test statistics and P-values for the non-significant predictors were computed by including them one by one into the final models. Random effect is given in SD ± 95% confidence interval. The estimated length of the eggs in the ‘predator-cue’ environment was 3.17 mm [3.10–3.25] and their estimated width was 2.21 mm [2.14–2.27] (with SVL and time of trial centered on their mean). Both measures increased with SVL (length: by 0.03 mm[0.01–0.06] per SVL unit, width: by 0.03 mm [0.004–0.05] per SVL unit), and both length and width of the eggs in the ‘no predator-cue’ environment also decreased with time compared to eggs in the ‘predator-cue’ environment (length: by -0.01 mm [-0.02–-0.003] per time unit, width: by -0.01 mm [-0.01–-0.001] per time unit). (DOCX) [file pone.0136044.s004.docx]

**S4 Table.** Test statistics and significance of the investigated predictors from the same models fitted to the original measures of the eggs (length and width) as to the first component of PCA on egg size. Final models are in bold; test statistics and *P*-values for the non-significant predictors were computed by including them one by one into the final models. Random effect is given in SD ± 95% confidence interval. The estimated length of the eggs in the ‘predator-cue’ environment was 3.17 mm [3.10-3.25] and their estimated width was 2.21 mm [2.14-2.27] (with SVL and time of trial centered on their mean). Both measures increased with SVL (length: by 0.03 mm[0.01-0.06] per SVL unit, width: by 0.03 mm [0.004-0.05] per SVL unit), and both length and width of the eggs in the ‘no predator-cue’ environment also decreased with time compared to eggs in the ‘predator-cue’ environment (length: by -0.01 mm [-0.02- -0.003] per time unit, width: by -0.01 mm [-0.01- -0.001] per time unit).

| **Model** | **Response variable** | **Random effect (‘Identity’)** | **Predictors** | **df** | **F** | **P** |
| --- | --- | --- | --- | --- | --- | --- |
| **LMM** | **Length of eggs** | **0.11 [0.05-0.23]** | **SVL** | **1,39** | **6.15** | **0.018** |
|  |  |  | **Time** | **1,39** | **10.18** | **0.003** |
|  |  |  | **Environment** | **1,40** | **0.55** | **0.461** |
|  |  |  | **Time × Environment** | **1,40** | **6.91** | **0.012** |
|  |  |  | Pond | 3,36 | 1.66 | 0.192 |
|  |  |  | Pond × Environment | 3,37 | 0.81 | 0.498 |
| **LMM** | **Width of eggs** | **0.13 [0.09-0.20]** | **SVL** | **1,39** | **4.88** | **0.033** |
|  |  |  | **Time** | **1,39** | **6.17** | **0.017** |
|  |  |  | **Environment** | **1,40** | **1.27** | **0.267** |
|  |  |  | **Time × Environment** | **1,40** | **5.31** | **0.026** |
|  |  |  | Pond | 3,36 | 0.51 | 0.681 |
|  |  |  | Pond × Environment | 3,37 | 2.17 | 0.108 |
